# Gene targeting in amyotrophic lateral sclerosis using causality-based feature selection and machine learning

**DOI:** 10.1186/s10020-023-00603-y

**Published:** 2023-01-24

**Authors:** Kyriaki Founta, Dimitra Dafou, Eirini Kanata, Theodoros Sklaviadis, Theodoros P. Zanos, Anastasios Gounaris, Konstantinos Xanthopoulos

**Affiliations:** 1 Donald and Barbara Zucker School of Medicine at Hofstra/Northwell, Northwell Health, Hempstead, NY 11549 USA; 2 Institute of Molecular Medicine, Feinstein Institutes for Medical Research, Northwell Health, Manhasset, NY 11030 USA; 3 Laboratory of Pharmacology, School of Pharmacy, School of Health Sciences, Aristotle University of Thessaloniki, 54124 Thessaloniki, Greece; 4 Department of Genetics, Development and Molecular Biology, School of Biology, Aristotle University of Thessaloniki, 54124 Thessaloniki, Greece; 5 Feinstein Institutes for Medical Research, Institute of Health Systems Science, Northwell Health, Manhasset, NY 11030 USA; 6 Institute of Bioelectronic Medicine, Feinstein Institutes for Medical Research, Northwell Health, Manhasset, NY 11030 USA; 7 School of Informatics, Aristotle University of Thessaloniki, 54124 Thessaloniki, Greece; 8 Institute of Applied Biosciences, Centre for Research and Technology Hellas, 57001 Thermi, Greece

**Keywords:** Gene expression, Causality-based feature selection, Dimensionality reduction, Machine learning

## Abstract

**Background:**

Amyotrophic lateral sclerosis (ALS) is a rare progressive neurodegenerative disease that affects upper and lower motor neurons. As the molecular basis of the disease is still elusive, the development of high-throughput sequencing technologies, combined with data mining techniques and machine learning methods, could provide remarkable results in identifying pathogenetic mechanisms. High dimensionality is a major problem when applying machine learning techniques in biomedical data analysis, since a huge number of features is available for a limited number of samples. The aim of this study was to develop a methodology for training interpretable machine learning models in the classification of ALS and ALS-subtypes samples, using gene expression datasets.

**Methods:**

We performed dimensionality reduction in gene expression data using a semi-automated preprocessing systematic gene selection procedure using Statistically Equivalent Signature (SES), a causality-based feature selection algorithm, followed by Boosted Regression Trees (XGBoost) and Random Forest to train the machine learning classifiers. The SHapley Additive exPlanations (SHAP values) were used for interpretation of the machine learning classifiers. The methodology was developed and tested using two distinct publicly available ALS RNA-seq datasets. We evaluated the performance of SES as a dimensionality reduction method against: (a) Least Absolute Shrinkage and Selection Operator (LASSO), and (b) Local Outlier Factor (LOF).

**Results:**

The proposed methodology achieved 85.18% accuracy for the classification of cerebellum or frontal cortex samples as *C9orf72*-related familial ALS, sporadic ALS or healthy samples. Importantly, the genes identified as the most determinative have also been reported as disease-associated in ALS literature. When tested in the evaluation dataset, the methodology achieved 88.89% accuracy for the classification of sporadic ALS motor neuron samples. When LASSO was used as feature selection method instead of SES, the accuracy of the machine learning classifiers ranged from 74.07 to 96.30%, depending on tissue assessed, while LOF underperformed significantly (77.78% accuracy for the classification of pooled cerebellum and frontal cortex samples).

**Conclusions:**

Using SES, we addressed the challenge of high dimensionality in gene expression data analysis, and we trained accurate machine learning ALS classifiers, specific for the gene expression patterns of different disease subtypes and tissue samples, while identifying disease-associated genes.

**Supplementary Information:**

The online version contains supplementary material available at 10.1186/s10020-023-00603-y.

## Introduction

Amyotrophic lateral sclerosis is a fatal neurodegenerative disease that affects motor pathways of both upper and lower motor neurons (Mathis et al. 2019; Ragagnin et al. 2019). Motor symptoms include progressive muscle atrophy and weakness that lead to paralysis and eventually death (Mejzini et al. 2019). There is also a wide range of non-motor symptoms, such as cognitive and behavioral changes, disruption of executive functions, frontotemporal dementia, and others (Goldstein and Abrahams 2013; Phukan et al. 2007; Volk et al. 2018). The disease can be either inherited or sporadic in origin, while clear pathogenetic causes have been so far identified in 40–55% of inherited ALS cases (Mejzini et al. 2019). As a result, in the vast majority of ALS cases the disease etiology remains unclear (Mathis et al. 2019; Volk et al. 2018), while clinical and basic research data suggest involvement of multiple genetic factors (Mathis et al. 2019). Most familial ALS cases are inherited in an autosomal dominant manner, accounting for ~ 10% of all ALS cases, while the remaining 90% are classified as sporadic ALS (Mejzini et al. 2019). Mutations in more than 30 genes and loci have been associated with ALS, including *SOD1*, *TARDBP*, *FUS*, and *C9orf72* (Nakamura et al. 2020). Although these genes are strongly associated with familial ALS, mutated forms have also been identified in a small percentage of sporadic ALS cases (Es et al. 2009). Nevertheless, a direct pathogenetic link between these gene mutations and the emergence of motor neuron degeneration has not yet been established (Mejzini et al. 2019). As a result, despite several disease-associated mutations have been identified, their relative importance in ALS pathogenesis remains unclear (Lederer et al. 2007).

In recent years, the advancements in high-throughput sequencing technologies have opened new perspectives in the elucidation of the underlying pathogenetic mechanisms of ALS. A plethora of whole genome association studies has resulted in the identification of ALS susceptibility loci and ALS-associated single nucleotide polymorphisms (SNPs) (Nakamura et al. 2020; Es et al. 2009; Sha et al. 2009), while gene expression studies have led to gene expression profiling of various tissues of the nervous system (Batra et al. 2016; Pantelidou et al. 2007; Prudencio et al. 2015). RNA-sequencing data has been used for a wide range of analyses, including weighted gene co-expression network analysis and protein–protein interaction networks analysis (Kotni et al. 2016; Saris et al. 2009) as well as unsupervised clustering analysis of gene expression for the molecular classification of ALS samples (Aronica et al. 2015; Morello et al. 2018). In this context, a classification pipeline based predominantly on machine learning techniques was recently introduced (Karim et al. 2021). However, the classification efficiency of machine learning (ML) methods deteriorates when applied to biomedical data; due to "*the curse of dimensionality"* (Vasilopoulou et al. 2020), exacerbated by the lack of large, labeled datasets to properly train the ML models*.* High dimensionality is a very common problem in such studies, since datasets include an extremely high number of features with a reduced number of samples (Vasilopoulou et al. 2020*).* Recently, an indirect solution to the problem was proposed, based on conversion of RNA expression data into images and use of these images to train a convolution neural network (CNN) (Karim et al. 2021). The main shortcoming of this approach is that the training data is too small to allow for efficient model building and separation between relevant and irrelevant genes. In other studies, a manual or ad-hoc selection process was used (Magen et al. 2020). In the meanwhile, AI-based approaches have started being considered as innovative gene targeting tools, employed recently in drug target discovery studies (Eisenstein 2022).

In this study, we introduce a novel semi-automated preprocessing gene selection methodology using SES, a causality-based feature selection algorithm that is tailored to high-dimensional datasets with a low number of samples. We used novel approaches to (i) achieve dimensionality reduction of gene expression data, while ensuring that genes with causality relationship and not mere statistical correlation will be retained; and (ii) train highly efficient interpretable classification models using XGBoost and Random Forest. For the interpretation of XGBoost models, we used the SHAP values (Shapley additive explanations). Our methodology was developed and evaluated using 2 publicly available ALS-related datasets and benchmarked against other gene selection approaches (Least Absolute Shrinkage and Selection Operator (LASSO) and Local Outlier Factor (LOF)), achieving high classification accuracy and model explanation efficacy, especially in more complex datasets. Of note, the genes selected through our methodology as the most determinative for disease existence have already been reported to play a role in ALS; this provides strong insights into the capability of our methodology not only to lead to better detection and prediction ML models but also assist in unraveling disease-associated genes.

## Data and methods

### Data

Two datasets were used in this study, as outlined in Table 1. For the development of the proposed methodology, we used RNA-sequencing data from the study of Prudencio et al. (Prudencio et al. 2015) which included: (a) cerebellum and frontal cortex post-mortem samples from 8 *C9orf72*-related familial ALS cases (8 frontal cortex and 8 cerebellum samples), (b) cerebellum and frontal cortex post-mortem samples from 10 sporadic ALS cases (10 frontal cortex and 10 cerebellum samples), and (c) cerebellum and frontal cortex post-mortem samples from 8 healthy individuals plus one frontal cortex sample from an additional healthy individual (9 frontal cortex and 8 cerebellum samples). For this dataset (Prudencio et al. 2015), gene expression and differential expression analyses were performed, following a standard protocol (Ghosh and Chan 2016). Additionally, a publicly available gene expression dataset from the study of Batra et al. (2016) was used to evaluate the efficiency of our methodology. This dataset included gene expression data for 12 sporadic ALS motor neuron samples and 9 healthy-control motor neuron samples.

**Table 1:** Data summary

Development dataset	A) Cerebellum and frontal cortex gene expression (logRPKM) data from 8 *C9orf72*-related familial ALS, 10 sporadic ALS and 9 healthy-control patients (for 1 of the healthy patients only frontal cortex expression data was available). In total, 53 cerebellum and frontal cortex samples B) Same data formatted as differential expression datasets for the sporadic ALS and the *C9orf72*-related familial ALS subtype (1 differential expression dataset per disease subtype)
Evaluation dataset	Spinal motor neuron gene expression (logRPKM) data from 12 sporadic ALS patients and 9 healthy-control patients

### Methodology

The SES algorithm is a causality-based feature selection algorithm, established on the principles of constrained-based learning of Bayesian Networks, from the “MXM” R package. SES follows a forward–backward filter approach for feature selection while providing minimal, highly-predictive, statistically-equivalent, multiple feature subsets of a high dimensional dataset (Anna Roumpelaki 2022). Using SES, we managed to develop a systematic gene selection process and successfully achieved dimensionality reduction by identifying the genes that have the highest probability to be causally related with ALS. After identifying the minimal set of features with the highest predictive performance on the target variable of interest (disease state: ALS or healthy), the SES algorithm uses these features to build regression models. The selected features can be further used to train disease classifiers.

**Fig. 1:**
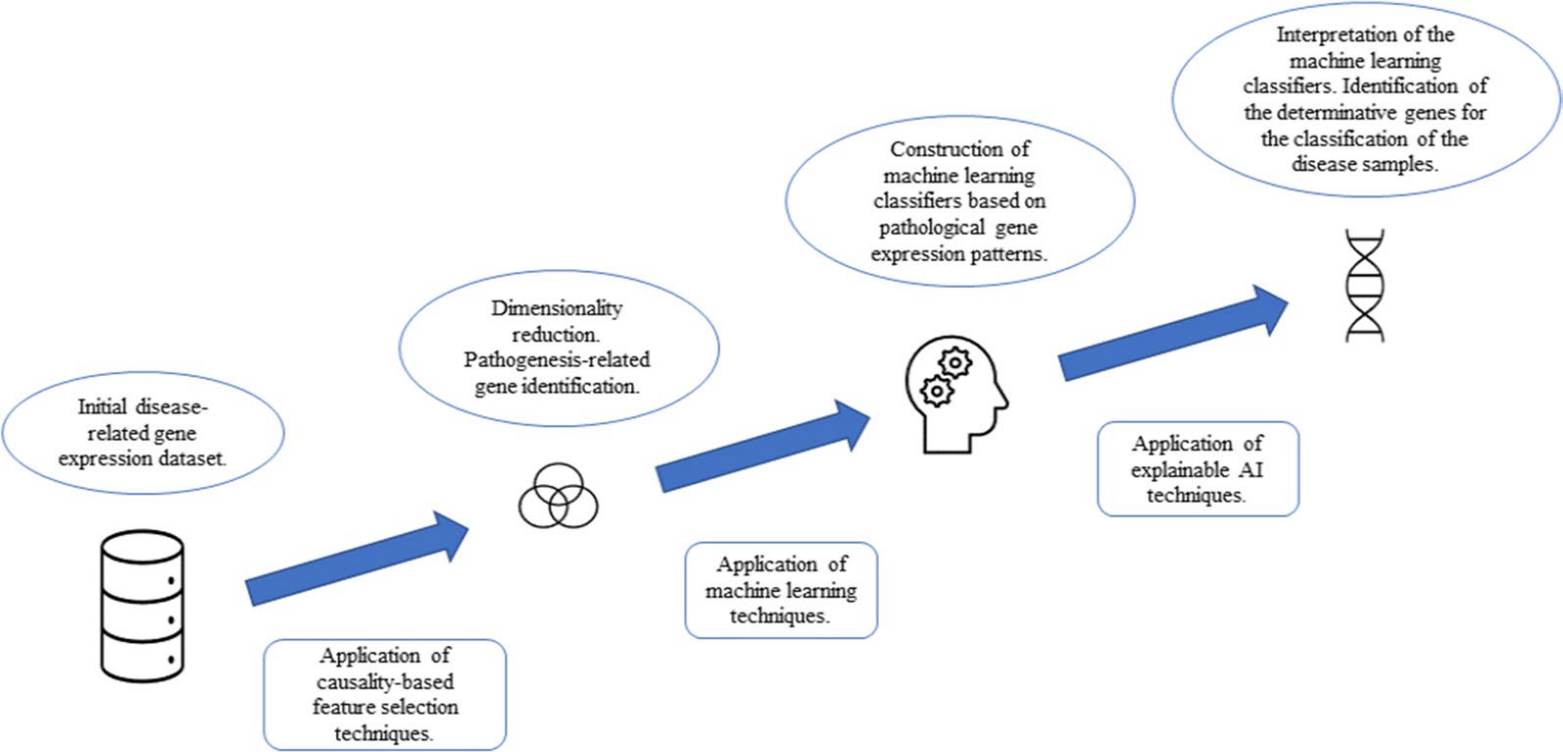
Schematic of the developed methodology

The newly-developed pipeline starts with application of the SES algorithm to detect the genes that constitute a subset of the Markov Blanket (parents and children only) of the target variable of interest (disease state -ALS or healthy- of a sample). Then machine learning classifiers are trained using the selected genes by applying the two tree-based machine learning algorithms XGBoost (“xgboost” R package) (Chen et al. 2016) and Random Forest (“randomForest” R package) (Breiman 2001). The classifiers are interpreted according to their SHAP values (“shapr” package). The proposed pipeline allows detection of the genes that are the determinative for the classification of a sample as diseased or healthy, emphasizing on causal relationships rather than statistical correlations that may be present in the data available (Fig. 1).

#### Feature selection with SES

The focus of our study is to identify the genes forming the optimal SES regression models. We used the SES algorithm to identify the highly-predictive subsets of genes, allowing discrimination between: (a) *C9orf72*-related familial ALS and healthy-control samples, (b) sporadic ALS and healthy-control samples, and (c) all ALS (both sporadic and *C9orf72*-related familial ALS) and healthy-control samples. Since datasets from two distinct anatomical regions of the brain were available (frontal cortex and cerebellum), 6 different binary class separation cases were performed.

SES was applied in a ninefold leave-out stratified cross-validation manner for 3 different *p-values* (0.01, 0.03, 0.05). We used various *p-values* to render the gene selection process as systematically as possible and minimize the possibility of omitting highly predictive genes due to use of an incorrect combination of parameters. The maximum number of equally-predictive regression models produced per algorithm repetition is defined by the *nsignat* parameter, while each regression model represents the selection of a different subset of genes-features from the initial gene expression data. In our case, the *nsignat* was set to 10, meaning that SES returned a maximum of 10 equivalent regression models per repetition.

#### Development of machine learning classifiers with XGBoost and Random Forest

Next, we used two tree-based machine learning algorithms, XGBoost and Random Forest, to train classifiers. The machine learning algorithms were trained on: (a) the expression values of the selected genes, (b) the initial high-dimensional gene expression data. Training the machine learning algorithms on the initial dataset (full dimensionality) aimed at checking whether the performed feature selection process was efficient in terms of the resulting ML model accuracy. Classifiers trained on the complete initial gene expression dataset are expected to have lower accuracy than those trained on the more restricted dataset. To generate dependable and accurate results, the same k-fold cross-validation process, as in the feature selection process, was followed in this step of the analysis.

#### Model interpretation with the SHAP values

Interpreting machine learning models with the SHAP (SHapley Additive exPlanations) values (Norsk Regnesentral and Package 2021) is an application of the emerging field of XAI (eXplainable Artificial Intelligence) (Barredo Arrieta et al. 2020). The SHAP values provide a way to rank the predictors by their importance in the model, as they represent the effect of each of the predictive features (in our case genes) on the predicted outcome (class prediction). To this end, we used the “shapr” package for the interpretation of the most accurate machine learning classifiers.

#### Evaluation methods

We first evaluated our methodology by applying it in data from another publicly available gene expression dataset (Batra et al. 2016), which included gene expression values from 12 sporadic ALS and 9 healthy-control motor neuron samples. Following the same process, we applied the SES algorithm in a ninefold leave-out stratified cross-validation approach for 3 different p-values (0.01, 0.03, 0.05) and the maximum number of SES regression models to be produced per run (*nsignat* parameter) was set to 10.

To further evaluate our methodology, we compared the gene selection performance of SES to that of LASSO. LASSO is a state-of-the-art feature selection algorithm, which focuses on correlations between predictive features and the target variable rather than causal relationships between them (Lagani et al. 2017; Yu et al. 2020). In this context, we used LASSO instead of SES and analyzed both the development gene expression (Prudencio et al. 2015) and the evaluation dataset (Batra et al. 2016). To simulate the multi-stage analysis performed with SES, we applied LASSO in a tenfold leave-out cross-validation manner for each dataset and we performed 50 repetitions (seeds 1–50) per fold.

Finally, we compared the feature selection performance of SES to that of a simple outlier detection process. To this end, we used the differential expression dataset we retrieved through differential expression analysis of the Prudencio et al. dataset (Prudencio et al. 2015). The method used for the outlier detection was the LOF algorithm, a density-based algorithm from “DMwR” R package (Torgo 2017). Sporadic ALS and *C9orf*72-related familial ALS differential expression datasets were merged and the common outliers between these two disease subtypes were identified.

## Results

### Gene selection and classifier construction

The genes selected during the multi-stage systematic feature selection process with SES carry all the necessary information for further classification of an unknown cerebellum or frontal cortex sample into different categories. When analyzing the development dataset, we were interested in the *C9orf72*-related familial ALS, sporadic ALS or healthy, 3-classification problem. While the development dataset included 23,188 genes, the genes selected by the systematic gene selection process with SES (the genes forming the multiple regression models for the 6 different binary class separation cases) were reduced to 473 (approx. 2%).

These 473 selected genes represent the first set of selected genes, a rather general set of genes for molecular detection of all the ALS cases (*C9orf72*-related familial ALS and sporadic ALS cases). However, if we assess the class separation accuracy achieved by the regression models of SES, we can identify those regression models (i.e., these highly-predictive subsets of genes) that are very accurate (thus more informative) for a specific molecular segregation case.

Table 2 displays the accuracy achieved by the SES regression models in the six different separation cases for the three different p-values when taking into consideration: (a) the first 3 out of 10 SES models, (b) the first 5 out of 10 SES models and (c) all 10 SES models.

**Table 2:** Comparison of performance of the SES models with different hyperparameters in the development dataset

	**p-value = 0.05**	**p-value = 0.03**	**p-value = 0.01**
**Cerebellum samples**			
*Separation of ALS (both C9orf72-related familial ALS and sporadic ALS) from healthy samples*			
10 models	74.11%	*81.48%*	77.77%
5 models	66.66%	81.48%	*85.18%*
3 models	74.88%	*77.77%*	70.37%
*Separation of C9orf72-related familial ALS from healthy samples*			
10 models	50.00%	33.33%	*66.66%*
5 models	72.22%	66.66%	*83.33%*
3 models	*72.22%*	*72.22%*	66.66%
*Separation of sporadic ALS from healthy samples*			
10 models	33.33%	22.22%	*38,88%*
5 models	33.33%	22.22%	*44.44%*
3 models	*33.33%*	22.22%	*33.33%*
**Frontal Cortex samples**			
*Separation of ALS (both C9orf72-related familial ALS and sporadic ALS) from healthy samples*			
10 models	48.14%	*51.85%*	*51.85%*
5 models	51.85%	51.85%	*55.55%*
3 models	51.85%	48.14%	*55.55%*
*Separation of C9orf72-related familial ALS from healthy samples*			
10 models	61.11%	*72.22%*	38.88%
5 models	*61.11%*	55.55%	44.44%
3 models	*55.55%*	50.00%	50.00%
*Separation of sporadic ALS from healthy samples*			
10 models	*61.11%*	*61.11%*	*61.11%*
5 models	55.55%	*61.11%*	55.55%
3 models	*55.55%*	*55.55%*	50.00%

Accuracy achieved by the SES regression models in the hold-out datasets for the separation of: (i) ALS (both C9orf72-related familial ALS and sporadic ALS) cerebellum from healthy cerebellum samples, (ii) C9orf72-related familial ALS cerebellum samples from healthy cerebellum samples, (iii) sporadic ALS cerebellum from healthy cerebellum samples, (iv) ALS (both C9orf72-related familial ALS and sporadic ALS) frontal cortex from healthy frontal cortex samples, (v)) C9orf72-related familial ALS frontal cortex samples from healthy frontal cortex samples, (vi) sporadic ALS frontal cortex from healthy frontal cortex samples. The referred accuracy is achieved due to taking into consideration: (a) the first 3 out of 10 SES models, (b) the first 5 out of 10 SES models and (c) all 10 SES models. The most accurate separation cases are highlighted in italics

Across the board (Table 2), SES regression models perform better in the separation cases of the cerebellum samples: by using the first 5 SES models for *p-*value = *0.01* we can distinguish if a cerebellum sample is an ALS (*C9orf72*-related familial ALS or sporadic ALS) or a healthy sample with 85.18% accuracy. Similarly, Table 2 also shows that by using the first 5 SES models for *p-*value = 0.01, we can identify an unknown ALS cerebellum sample as a *C9orf72*-related familial ALS sample with 83.33% accuracy. Consequently, these 44 genes that constitute these “5 plus 5” regression models, represent a second, more focused set of selected genes, specifically associated with the cerebellum samples. In contrast, the frontal cortex SES separation models were generally less accurate, reaching 72.22% accuracy, when *p-value* and *nsignat* were set at 0.03 and 10 respectively. For that reason, we decided not to focus on gene subsets specifically associated with the frontal cortex samples.

A ninefold leave-out stratified cross-validation process was then followed by training machine learning classifiers on: (a) the expression values of the 473 genes, (b) the expression values of the 44 genes, and (c) the expression values of the 23,188 genes (initial gene expression dataset). The accuracy results for the classifiers for the 3-class classification (*C9orf72*-related familial ALS, sporadic ALS or healthy) of the unknown (hold-out) samples are presented below (Table 3).

**Table 3:** Comparison of model performance trained on different numbers of selected genes

	473 genes	44 genes	Full dimensionality
*XGBoost classifiers*			
Pooled cerebellum and frontal cortex samples	*72.33%*	67.02%	44.25%
Cerebellum samples	70.37%	*85.18%*	48.14%
Frontal cortex samples	*85.18%*	81.48%	51.85%
*Random Forest classifiers*			
Pooled cerebellum and frontal cortex samples	74.22%	*79.75%*	46.96%
Cerebellum samples	37.03%	*40.74%*	25.92%
Frontal cortex samples	29.62%	*40.74%*	25.92%

Accuracy achieved by the XGBoost and Random Forest classifiers for the classification of the hold-out datasets when they were trained on (a) the expression values of the 473 geness, (b) the expression values of the 44 genes, (c) full dimensionality (expression values of 23,188 genes). The analysis was performed for (i) pooled cerebellum and frontal cortex samples, (ii) only for the cerebellum samples, as well as (c) only for the frontal cortex samples. The most accurate classification cases are highlighted in italics

The machine learning models trained on the initial high-dimensional gene expression dataset (23,188 genes) achieved lower classification accuracy in comparison to those trained on the 473 or the 44 genes. Moreover, Random Forest performed better than XGBoost at building machine learning classifiers in pooled cerebellum and frontal cortex samples and less so when the two brain regions were analyzed separately (Table 3).

SHAP values were calculated, and the results were plotted as SHAP importance plots, which visualize the level of importance of the 10 most determinative genes for each classifier. As we had followed a ninefold stratified cross-validation analysis, 9 cerebellum and 9 frontal cortex classifiers, 9 SHAP importance plots for the interpretation of the cerebellum classifiers and 9 SHAP importance plots for the interpretation of frontal cortex classifiers were made. The mean average importance score for each gene appearing in these SHAP explanation plots was computed (Additional file 1: Table S1). For both cerebellum and frontal cortex classifiers the *C9orf72* gene was identified as one of the most determinative for the classification of an ALS sample to the specific *C9orf72*-related familial ALS subtype.

### Methodology evaluation in a different ALS dataset

Feature selection with SES was also performed for the evaluation dataset and the assessed accuracy achieved by the regression models in distinguishing sporadic ALS from healthy samples was computed (Additional file 1: Table S2).

The number of the different genes identified in at least one regression model during the whole process was 93 out of the 24,626 included in the dataset (approx. 0.37% of the genes were retained in the analysis). This subset of the 93 selected genes could be characterized as the first, more generalized set of informative genes. We then focused on the regression models that were constructed during 9-folds with *p-*value = 0.01, which achieved the highest mean average ninefold accuracy among the others. The genes selected by these models were 19.

For the construction of the machine learning classifiers, we chose to use these 19 genes. A ninefold leave-out stratified cross-validation analysis was performed by applying XGBoost and Random Forest, generating highly accurate machine learning models, trained upon the 19 genes. In this case, XGBoost and Random Forest classifiers achieved 88.89% accuracy for the 2-class classification (sporadic ALS or healthy motor neuron samples) of the hold-out datasets, while the respective accuracy achieved by the classifiers that were trained upon all features of the gene expression dataset (24,626 genes) was 33.33%. The SHAP values for the interpretation of the motor neuron classifiers and the detection of the most determinative genes for the classification of an unknown sample as sporadic ALS or healthy were also computed (Additional file 1: Table S2).

### Replacing SES with LASSO

When SES was replaced by LASSO in the pipeline, 99 genes were selected for the first, more generalized set of informative genes, and 39 for the more accurate subset of informative genes. Similar to our previous results, the XGBoost classifiers performed better than the Random Forest classifiers. Interestingly, the cerebellum XGBoost classifiers trained upon the LASSO-selected genes achieved 96.3% classification accuracy in the 3-class classification (*C9orf72*-related familial ALS, sporadic ALS, or healthy) of the hold-out datasets, while the frontal cortex XGBoost classifiers were only 74.07% accurate (Table 4).

**Table 4:** Comparison of model performance of two dimensionality reduction methods

	LASSO genes	SES genes
*XGBoost classifiers*		
Cerebellum classifiers	96.30%	85.18%
Frontal cortex classifiers	74.07%	85.18%
Motor neuron classifiers	88.89%	88.89%
*Random Forest classifiers*		
Cerebellum classifiers	40.74%	40.74%
Frontal cortex classifiers	37.04%	29.63%
Motor neuron classifiers	100.00%	88.89%

Achieved accuracy of the cerebellum and frontal cortex classifiers (derived from analyzing the development dataset), as well as the motor neuron classifiers (derived from analyzing the evaluation dataset). The respective accuracy is presented for the classifiers that were trained on the genes selected (a) by LASSO, and (b) by SES

Analysis of the evaluation dataset with LASSO led to the identification of 14 genes as the most predictive (second, more accurate, set of informative genes). When these 14 genes were used to train machine learning models, both Random Forest and XGBoost performed well and managed to train highly accurate classifiers, with Random Forest classifiers achieving up to 100% accuracy in the class prediction of the hold-out datasets (Table 4). SHAP values were calculated for the interpretation of the classifiers trained on the LASSO-selected genes.

### Replacing SES with density-based outlier detection

Using the LOF algorithm for outlier identification in the differential expression data, we detected 199 outlier genes. These 199 genes are the common most differentially expressed genes between all ALS cases (both sporadic ALS and C9orf72-related familial ALS) and were used to train machine learning classifiers. The XGBoost classifiers trained on the LOF selected genes achieved 77.78% mean average accuracy in the 3-class classification (sporadic ALS, *C9orf72*-related familial ALS or healthy) of the hold-out datasets, while the achieved accuracy for the Random Forest classifiers was 48.15%.

**Fig. 2:**
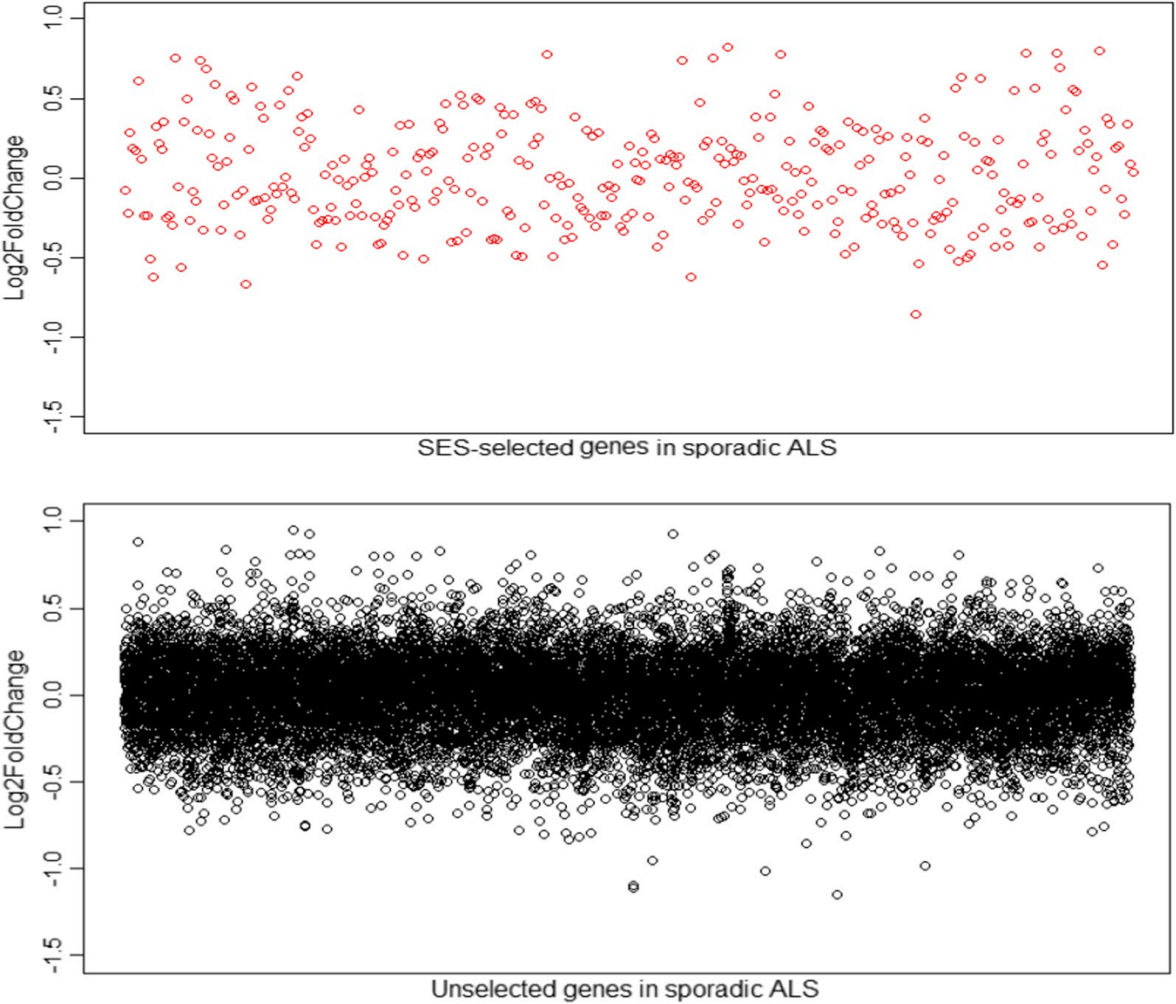
Differential expression levels (Log2 fold-change values) of the SES-selected genes in the sporadic ALS differential expression dataset (top panel), and the genes that were not selected by SES in the sporadic ALS differential expression dataset (bottom panel)

**Fig. 3:**
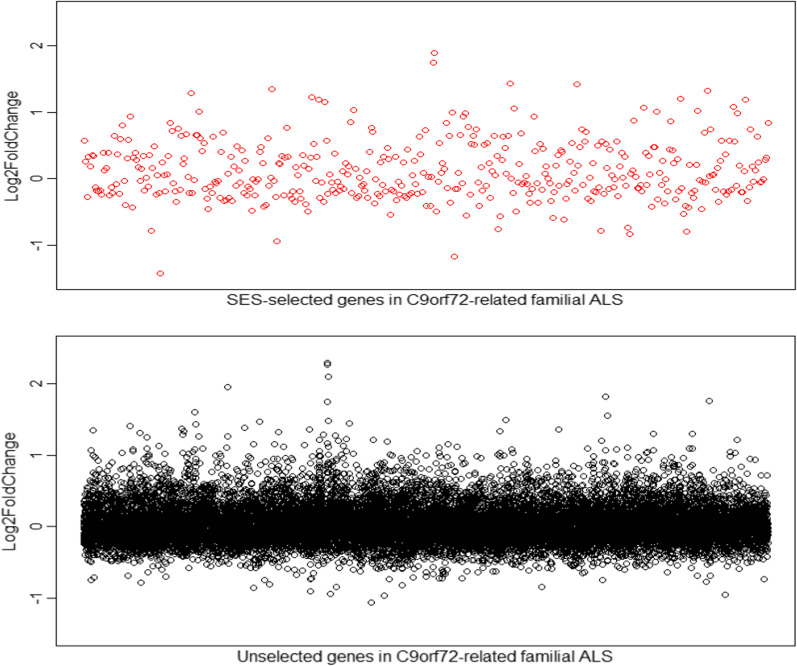
Differential expression levels (Log2 fold-change values) of the SES-selected genes in the *C9orf72-related* familial ALS differential expression dataset (top panel), and the genes that were not selected by SES in the *C9orf72-related* familial ALS differential expression dataset (bottom panel)

Comparing the results obtained, it becomes evident that the XGBoost classifiers trained on the 199 LOF selected genes have lower accuracy than those trained by the genes selected by SES. This indicates that the expression of some genes that are very informative for disease prediction does not change significantly and as a result these genes are not identified by simple outlier detection but are identified by SES. Figures 2 and 3 depict the differential expression of the genes in the sporadic and the *C9orf72*-related familial ALS differential expression dataset respectively. The SES-selected genes are shown in red and although the expression of some changes significantly, only minimal differences in the differential expression levels are found between them and the rest of the genes that were not selected by the application of the SES algorithm (Figs. 2, 3).

## Discussion

Taking advantage of gene expression data is often hampered by the high dimensionality that inevitably such datasets carry. At the same time, the molecular aspects of diseases like ALS remain elusive and research would benefit greatly by integration of such data. In this study, we developed an innovative methodology to identify a subset of causally related genes and then use it to train machine learning algorithms to efficiently differentiate between ALS subtypes and healthy-control samples.

By applying the causality-based feature selection algorithm SES, we managed to overcome the problem of high-dimensionality in the gene expression datasets, by only selecting the most informative genes for the disease prediction. Additionally, we used these genes to build highly accurate machine learning classifiers for the development dataset using XGBoost and Random Forest algorithm. High classification accuracy was also achieved when the same methodology was applied for the construction of ALS classifiers for the evaluation dataset.

When constructing the machine learning classifiers for the development dataset we observed that: (1) Random Forest classifiers could not perform optimally in the 3-class classification task (healthy sample, sporadic ALS sample or *C9orf72*-related ALS sample) when cerebellum and frontal cortex samples were studied separately, while the classifiers for pooled cerebellum and frontal cortex samples achieved high accuracy, and (2) the optimal number of genes (features) for the training datasets of the XGBoost cerebellum classifiers (expression values of 44 genes) proved to be lower than that of the XGBoost frontal cortex classifiers (expression values of 473 genes). The first observation should be mostly attributed to the smaller size of the training datasets when the two brain regions are studied separately in comparison to the training size when cerebellum and frontal cortex samples are pooled (23 and samples per training dataset for the cerebellum and the frontal cortex classifiers respectively, versus 47 samples per training dataset for pooled cerebellum and frontal cortex classifiers). However, in the analysis of the evaluation dataset, which also included a small number of samples (12 sporadic ALS and 9 healthy samples) the Random Forest algorithm performed well (88.89% classification accuracy). Based on these results, both the small size of the training dataset and the task complexity seem to lead to underperformance of the Random Forest classifiers. A small training dataset proved not to be sufficient for solving the 3-class classification problem in the development dataset, while a training dataset of a similar size was suitable for training binary classifiers for the evaluation dataset. Moreover, the fact that the optimal number of genes (features) in the training datasets of the frontal cortex classifiers is bigger than that of the cerebellum classifiers might be associated with the respective difference in the gene expression patterns between these two brain regions. More specifically, the cerebellum displays the least internal heterogeneity in gene expression patterns among all brain regions (Negi et al. 2017). Such information is in accordance with our findings indicating that higher heterogeneity in the brain region, requires a higher number of variables (genes) to train accurate machine learning models for the classification of its samples. In contrast, more homogenous brain regions seem to require fewer features for the classification of their samples. Specifically for the cerebellum classifiers, an increase in the dimensionality of the training datasets led to a decrease in model performance: the accuracy achieved by the XGBoost classifiers when they were trained on the 473 selected genes was ≈15% lower than that achieved when they were trained on the 44 genes (Table 3), which may imply fact that additional predictive variables, other than the absolutely necessary only add noise. As a final step in our methodology, we interpreted the best XGBoost classifiers by applying the SHAP values.

The methodology that we developed is among the first to take advantage of ML methods in order to provide insights into the molecular mechanisms associated specifically with ALS pathogenesis (Pun et al. 2022; Zhang et al. 2022; Bean et al. 2020). Throughout these analyses, we identified the genes most highly related to ALS pathogenesis for both sporadic ALS and *C9orf72*-related ALS cases, while performing separate analysis for cerebellum and frontal cortex samples (Table 2). Our main argument is twofold: firstly, through our proposed judicious gene selection procedure, the machine learning models are more accurate and secondly and more importantly, due to the causality-awareness of the feature selection methodology, the selected genes are more informative. In our experiments, we have mostly provided evidence regarding the former aspect. Regarding the latter, the most interesting observation associated with the SHAP interpretation results is that *C9orf7*, which has successfully been detected by our methodology as one of the most determinative genes for the existence of *C9orf72*-related ALS, was not the only one among the detected determinative genes that has already been defined as ALS-related. In fact, most of the genes identified as determinative, especially those with the highest mean average importance score, have already been associated with ALS or implicated in neurodegeneration events.

If we applied a threshold of mean average importance score of 3 to select the topmost determinative genes among the ones identified (Additional file 1: Table S1), we would identify *LOC100506258*, *ELOA3C*, *TNFRSF11B*, *STAT3*, *DDO* as the topmost determinative genes for the classification of a cerebellum sample as ALS or healthy. Among these genes, three (*TNFRSF11B*, *STAT3*, *DDO)* have already been cited at least once in ALS literature (Errico et al. 2020; Rubino et al. 2020; Shibata et al. 2010). Especially for *TNFRSF11B* and *STAT3,* a strong association with ALS pathology has been observed, the exact mechanism of which remains unclear (Rubino et al. 2020; Shibata et al. 2010). As for the categorization of an ALS cerebellum sample to a specific ALS subtype (sporadic ALS or C*9orf72*-related ALS), the topmost determinative genes were *C9orf72* and *PRDM13*. *C9orf72* is widely accepted as the most common genetic cause of amyotrophic lateral sclerosis (Yang et al. 2020), while the *PRDM13* gene, although not yet related to ALS, plays a major role in neuron differentiation and maintenance by encoding a transcriptional repressor (Bessodes et al. 2017; Leszczyński et al. 2020).

Furthermore, the *SAP18* gene, with a mean average importance score of 5.33 for the ALS subtype categorization in frontal cortex samples (Additional file 1: Table S1), has been shown to be related to brain cell dysregulation and development of amyloid plaques in both Down Syndrome and Alzheimer’s disease (Sharma et al. 2021), while *BAG1,* also one of the topmost determinative genes for the ALS categorization, as a member of PQC system proteins, is highly expressed in muscle of TG-ALS mice (Cicardi et al. 2018). For the classification of an ALS frontal cortex sample to a specific ALS type (sporadic ALS or *C9orf72*-related ALS), apart from the *C9orf72* gene, among the topmost determinative genes was also *ATP2A1*, a gene associated with glucose metabolism, related to the downregulation of Muscular differentiation and proliferation in ALS (Silroy and Bhowal 2018).

In summary, these findings indicate that the proposed methodology allows not only detection of genes with diagnostic value, but also of genes with a role in disease pathogenesis.

Additionally, when the methodology was applied in the evaluation dataset highly accurate machine learning classifiers were constructed ("Gene selection and classifier construction" section), while the genes identified as determinative for the classification of a motor neuron sample as sporadic ALS or healthy (Additional file 1: Table S1) were widely referenced in ALS and neurodegeneration literature. More specifically, the topmost determinative genes (genes with mean average importance score >  = 3) for the existence of sporadic ALS in motor neuron samples were *ΑΝΧΑ5, DDB1, EPB41* and *PRUNE*. All 4 genes have already been associated with ALS pathology. It has already been proposed that apoptosis plays a crucial role in ALS neurodegeneration and the *ΑΝΧΑ5* gene encoding for Annexin A5, which is strongly involved in apoptosis and survival mechanisms, has been characterized as a candidate gene for ALS (Morello et al. 2017). Moreover, *DDB1* encodes *DNA Damage Binding protein 1*, which associates with *CUL4* protein to assemble an ubiquitin ligase. The *CUL4*/ *DDB1* ubiquitin ligase has been shown to participate in proteasomal degradation of *Nrf2*, a transcription factor that regulates the expression of genes involved in cellular protection against damage by oxidants, electrophiles and inflammatory agents. The function of *Nrf2* has been proved to be altered in ALS (Dinkova-Kostova et al. 2018; Higa and Zhang 2007). As for *EPB41*, the gene has been identified as one of the 3 ALS-decreased differentially expressed genes (DEGs) in blood nearest to susceptibility loci (along with *METTL21A* and *TIAM1*). This observation indicates that the under-expression of *EPB41* is not a simple marker of disease progression. The gene seems to be involved in disease-causing pathogenic mechanisms instead (Swindell et al. 2019). Finally, *PRUNE* has also been mentioned in ALS literature. More specifically, it has been detected to be one of the top 5 DEGs in Sensory Neurons of mutant SOD1 mice (Liu et al. 2020).

The fact that our methodology, in addition to detecting ALS-related genes, also reveals neurodegeneration-associated genes that have not yet been related to ALS pathology in a computationally efficient manner, further strengthens the value of our findings since it can guide future research and leads to identifying causative, underlying disease mechanisms. This is the main advantage of using the SES algorithm over conventional feature selection methods, like the LASSO algorithm (which has been proved to be dependent on the heterogeneity of the analyzing brain region) or simple outlier detection. Especially for the outlier detection case, we showed that a marked difference in the gene expression is not always sufficient for deciding whether this gene is determinative for disease diagnosis or not (Figs. 2, 3). As for the LASSO algorithm, our results indicate that the success of the feature selection process it performs varies, which is a sign of overfitting, and most probably depends on the expression patterns of the studied brain region (cerebellum XGBoost classifiers trained on the LASSO-selected genes outperformed cerebellum XGBoost classifiers trained on the SES-selected genes, while the exact opposite was observed for the frontal cortex classifiers). In contrast, the performance of the classifiers trained on the SES-selected genes seems to be independent of the brain region under study (Table 4).

In general, LASSO was able to perform equally well with SES in detecting highly-accurate gene subsets from high dimensional gene expression data for both gene expression datasets. However, LASSO-identified genes might not be as valuable as the genes selected by SES, as LASSO captures only correlations between features and the target variable, while SES focuses on detecting features that are causally related with the target variable. Furthermore, in all cases, the genes selected by LASSO were fewer than those selected by SES. However, we cannot argue whether the genes selected by SES are more widely varied than those selected by LASSO. If we wanted to make a fair comparison regarding the number of genes each algorithm selects, we should have also followed a tenfold 50-repetition process with SES (as we did with LASSO) by taking into account only the first SES regression model per repetition. In a similar study that performed the same comparisons (Lagani et al. 2017), it was shown that LASSO selects more varied genes than SES. This is expected, since LASSO retrieves a superset of the Markov Blanket of the target variable (the Markov blanket plus some additional noisy features), while SES detects a subset of it (the parents and children of the target variable) (Lagani et al. 2017). In general, the comparison between SES and LASSO requires more research and might be use-case specific. Selecting the optimal approach would involve, apart from the observed accuracy in specific models and datasets, several qualitative factors that cannot be easily assessed and/or quantified; nevertheless, when targeting statistical correlations rather than causal relationships, the selected genes are, based on our results, less capable to assist in understanding the disease deeper.

Therefore, we additionally used the SHAP values to interpret the classifiers trained on the LASSO-selected genes (Additional file 1: Table S4). For the classification of the *C9orf72*-related familial ALS and sporadic ALS cerebellum and frontal cortex samples (development dataset), most of the genes identified as determinative for the classifiers trained on LASSO genes, had also been detected as determinative for the classifiers trained on the SES-selected genes. However, in many cases genes that were ranked among the topmost determinative (m.a. importance score > 3) for the LASSO-selected-genes trained classifiers, detected to have noticeably lower importance (m.a. importance score =  < 3) for the SES-genes-trained classifiers.

After a thorough research of ALS literature, we found that the majority of the genes identified among the topmost determinative for the classifiers trained on the LASSO-selected genes had already been reported in the literature, either displaying abnormal expression patterns in ALS cases or participating in pathways that are disrupted when the disease occurs, due to dysfunction in another gene in the same pathway. The latter is the case for *DSCAM,* which has been ranked among the topmost determinative genes for the classification of a cerebellum sample as ALS or healthy-control by the LASSO-genes trained classifiers (Additional file 1: Table S4)*. DSCAM* encodes a cell-surface receptor, while a specific *DSCAM* isoform (*DscamTM2*) is involved in axon processes and DSCAM gain-of-function axon phenotypes have been reported to be suppressed in ALS due to dysfunction in protein VAP (Yang et al. 2012). Additionally, for the further classification of an ALS cerebellum sample as *C9orf72*-ALS or sporadic ALS by the classifiers trained on LASSO-selected genes, *HOXC10 was* identified as the second most determinative gene (ranked immediately after *C9orf72*) (Additional file 1: Table S4)*.* Differential expression of *HOXC10* in ALS has already been reported by several groups (Loffreda et al. 2020; Shtilbans et al. 2011).

On the other hand, for the classification of a frontal cortex sample as ALS or healthy, among the four topmost determinative genes identified for the classifiers trained on the LASSO-selected genes, aberrant expression of two of them has already been detected in neurological disorders. More specifically, *DDTL* overexpression has been reported as possibly being involved in the pathology of Schizophrenia (Nakamura et al. 2015), while increased levels of ACYP have been detected in Alzheimer's disease patients’ fibroblasts (DeglInnocenti et al. 2019). For the further classification of an ALS frontal cortex samples to a specific subtype (*C9orf72*-ALS or sporadic ALS), the genes identified as the topmost determinative for the LASSO-genes-trained classifiers had also been identified as important, but not among the topmost determinative, for the SES- genes-trained classifiers. *GP9* was one of those genes. In fact, *GP9* has already been associated with neural-dysregulation and neurotoxicity, as aberrant expression of this gene has been measured in blood cells of Epilepsy and Multiple Sclerosis patients (Li et al. 2022; Berge et al. 2019).

As for the motor neuron classifiers trained on the LASSO-selected genes, apart from *DDB1*, which had also been identified among the topmost determinative for the SES-genes-trained classifiers, the rest of the topmost determinative were uniquely associated with the LASSO-genes-trained classifiers. Interestingly, abnormal expression patterns for all of them have already been detected either in ALS patients [*MIR16* (Joilin et al. 2019; Liguori et al. 2018), *CCDC85B* (Miller et al. 2018)] or in Alzheimer’s Disease patients (*GJA5)* (Ziff et al. 2022).

These results further indicate that LASSO, like LOF, focuses on the outliers of the dataset when performing feature selection and thus, in our case, the genes with aberrant expression patterns were selected. In contrast, we have proven that SES doesn’t fully rely on outlier detection when performing feature selection (Figs. 2, 3). Based on the above, we can claim that our methodology is capable to better detect genes that are more likely to be involved more directly in the pathological events leading to ALS pathology, rather than simply identifying genes and pathways that are dysregulated once the disease has occurred.

## Conclusion

In conclusion, this study introduces a novel methodology aiming at reducing gene expression data dimensionality and identifying causal ALS genes that may be responsible for the occurrence of ALS in general, or of a specific ALS subtype (sporadic ALS or *C9orf72*-related familial ALS). Using these genes, more accurate machine learning classifiers that can diagnose ALS and its specific subtypes can be built. More importantly, the more informative genes are detected with low computational effort. In addition, our proposal can be deemed as a generic methodology that is applicable to other diseases as well.

## Supplementary Information

**Additional file 1.** Appendix containing Additional Tables S1–S4.

## Data Availability

Data analysed during this study are included in these published articles: Batra R, Hutt K, Vu A, Rabin SJ, Baughn MW, Libby RT, et al. Gene Expression Signatures of Sporadic ALS Motor Neuron Populations [Internet]. Neuroscience; 2016 Feb [cited 2022 Sep 6]. Prudencio M, Belzil VV, Batra R, Ross CA, Gendron TF, Pregent LJ, et al. Distinct brain transcriptome profiles in C9orf72-associated and sporadic ALS. Nat Neurosci. 2015 Aug;18(8):1175–82. The code generated for this study is available from the corresponding author on reasonable request.
