# Supplementary material for: Gene targeting in amyotrophic lateral sclerosis using causality-based feature selection and machine learning
Source: Mol Med. 2023 Jan 24;29:12. doi: 10.1186/s10020-023-00603-y (PMC9872307; doi:10.1186/s10020-023-00603-y)
Supplement: Supplementary file 1 — Additional file 1. Appendix containing Additional Tables S1–S4. [file 10020_2023_603_MOESM1_ESM.docx]

**Additional** Appendix

*SYSTEMATIC GENE SELECTION FOR ENHANCING INTERPRETABLE MACHINE LEARNING MOLECULAR CLASSIFIERS: THE CASE OF AMYOTROPHIC LATERAL SCLEROSIS*

By Founta *et al.*

**Additional Table S1. Classification determinative genes for the classifiers trained on the SES-selected genes.**

| **Cerebellum classifiers (44 genes analysis)** | | |
| --- | --- | --- |
| ***ALS or healthy sample*** | | |
|  | **Determinative gene** | **Mean average importance score** |
|  | *LOC100506258* | 7.11 |
|  | *ELOA3C* | 6.33 |
|  | *TNFRSF11B* | 5.56 |
|  | *STAT3* | 4.67 |
|  | *DDO* | 3.11 |
|  | *CRYZL1* | 3.00 |
|  | *CCDC157* | 2.89 |
|  | *SERPIND1* | 2.11 |
|  | *GDF10* | 1.78 |
|  | *KLHDC8A* | 1.67 |
|  | *LINC02011* | 1.67 |
|  | *CYP2E1* | 1.44 |
|  | *C1RL* | 1.11 |
|  | *CD27* | 1.00 |
|  | *CFAP57* | 0.89 |
|  | *NACAP1* | 0.78 |
|  | *APTR* | 0.67 |
|  | *TPST1* | 0.55 |
|  | *CRYBA1* | 0.44 |
| ***C9orf72-related familial ALS or sporadic ALS sample*** | | |
|  | *C9orf72* | 9.11 |
|  | *PRDM13* | 8.89 |
| **Frontal cortex classifiers (473 genes analysis)** | | |
| ***ALS or healthy sample*** | | |
|  | *SAP18* | 5.33 |
|  | *BAG1* | 3.44 |
|  | *LINC01574* | 3.11 |
|  | *FUNDC1* | 2.67 |
|  | *ALMS1P1* | 2.33 |
|  | *PPP1R3F* | 2.11 |
|  | *NOL7* | 2.11 |
|  | *ACYP2* | 1.78 |
|  | *MIR4477A* | 1.44 |
|  | *ATXN7L3* | 1.44 |
|  | *TMEM14C* | 1.33 |
|  | *MPLKIP* | 1.11 |
|  | *ARL6IP1* | 1.11 |
|  | *ATOH7* | 1.11 |
|  | *RRS1* | 1.00 |
|  | *COX7B* | 1.00 |
|  | *TMEM185A* | 0.89 |
|  | *RXRB* | 0.89 |
|  | *ARHGAP10* | 0.89 |
|  | *SSBP3* | 0.78 |
|  | *ANAPC13* | 0.78 |
|  | *ALAS2* | 0.66 |
|  | *SPATA33* | 0.66 |
|  | *IQSEC3* | 0.56 |
|  | *DPCD* | 0.56 |
|  | *TMED10* | 0.44 |
|  | *L3MBTL3* | 0.33 |
|  | *DDTL* | 0.33 |
|  | *CTNNBIP1* | 0.33 |
|  | *A2M.AS1* | 0.11 |
|  | *CHMP4B* | 0.0185 |
| ***C9orf72-related familial ALS or sporadic ALS sample*** | | |
|  | *LINC00658* | 5.44 |
|  | *HOXD9* | 4.56 |
|  | *C9orf72* | 4.00 |
|  | *ATP2A1* | 3.89 |
|  | *MPND* | 3.00 |
|  | *AGPAT5* | 3.00 |
|  | *DISC1* | 2.33 |
|  | *AKR7L* | 2.11 |
|  | *GP9* | 1.78 |
|  | *ALG8* | 1.00 |
|  | *AGAP12P* | 1.00 |
|  | *CFAP57* | 0.89 |
|  | *C9orf24* | 0.33 |
| **Motor neuron classifiers (19 genes)** | | |
| ***Sporadic ALS or healthy sample*** | | |
|  | *ΑΝΧΑ5* | 8.78 |
|  | *DDB1* | 4.44 |
|  | *EPB41* | 3.11 |
|  | *PRUNE* | 3.00 |
|  | *ARMC10* | 2.78 |
|  | *DFKZp761G1923* | 2.44 |
|  | *GOLGA7* | 2.33 |
|  | *CSTB* | 1.11 |

*Interpretation of the cerebellum (trained upon the 44 SES selected genes) and frontal cortex (trained upon the 473 SES selected genes) classifiers (dataset of Prudencio et al.), as well as the motor neuron (trained upon the 19 selected SES genes) classifiers (dataset of Batra et al.). The table displays the determinative genes for the classification of an unknown cerebellum and frontal cortex sample as a) ALS or healthy, and additionally for the identification of a b)* *C9orf72-related familial ALS or sporadic ALS sample. Moreover, the determinative genes are presented for classifying an unknown motor neuron sample as sporadic ALS or healthy.*

**Additional Table S2. Comparison of performance of the SES models with different hyperparameters for the 9-fold feature selection process in the evaluation dataset.**

|  | **p-value = 0.05** | **p-value = 0.03** | **p-value = 0.01** |
| --- | --- | --- | --- |
| Fold 1 | 95.00% | 50.00% | 66.67% |
| Fold 2 | 66.67% | 66.67% | 80.00% |
| Fold 3 | 90.00% | 90.00% | 100.00% |
| Fold 4 | 50.00% | 62.50% | 66.67% |
| Fold 5 | 100.00% | 100.00% | 100.00% |
| Fold 6 | 33.33% | 25.00% | 66.67% |
| Fold 7 | 50.00% | 50.00% | 50.00% |
| Fold 8 | 50.00% | 50.00% | 50.00% |
| Fold 9 | 62.50% | 64.29% | 50.00% |
| **Mean average 9-fold accuracy** | **66.00%** | **62.00%** | **73.00%** |

**Additional Table S3. Performance metrics for the best XGBoost classifiers for each of the three brain regions.**

|  | **Accuracy** | **Precision** | **Recall** | **MacroF1** | **MicroF1** |
| --- | --- | --- | --- | --- | --- |
| Cerebellum classifiers | 85.18% | 85.18% | 79.62% | 81.48% | 82.31% |
| Frontal cortex classifiers | 85.18% | 85.18% | 77.77% | 80.24% | 81.31% |
| Motor neuron classifiers | 88.88% | 93.75% | 88.88% | 95.83% | 91.25% |

**Additional Table S4. Classification determinative genes for the classifiers trained on LASSO-selected genes.** *Genes also identified as both important and among the topmost determinative (m.a. importance score > 3) for the classifiers trained on the SES-selected genes are highlighted in red, while genes identified as important, but not among the topmost determinative for the SES-genes trained classifiers are highlighted in blue.*

| **Cerebellum classifiers** | | |
| --- | --- | --- |
| ***ALS or healthy sample*** | | |
|  | **Determinative gene** | **Mean average importance score** |
|  | *ELOA3C* | 8.56 |
|  | *DSCAM* | 5.33 |
|  | *CRYZL1* | 4.67 |
|  | *CFAP57* | 4.33 |
|  | *C1RL* | 3.44 |
|  | *LINC02011* | 2.89 |
|  | *NACAP1* | 2.89 |
|  | *PTGES2.AS1* | 2.56 |
|  | *LRCC36* | 2.11 |
|  | *SNX9* | 2.00 |
|  | *TNFRSF11B* | 1.89 |
|  | *IGFL2* | 1.22 |
|  | *GDF10* | 0.89 |
|  | *CDH1* | 0.89 |
|  | *CPNE8* | 0.78 |
|  | *RRS1.AS1* | 0.56 |
|  | *RN7SL3* | 0.44 |
|  | *GTSE1* | 0.22 |
| ***C9orf72-related familial ALS or sporadic ALS sample*** | | |
|  | *C9orf72* | 9.11 |
|  | *HOXC10* | 8.89 |
| **Frontal cortex classifiers** | | |
| ***ALS or healthy sample*** | | |
|  | *LINC01574* | 6.11 |
|  | *ALMS1P1* | 5.33 |
|  | *DDTL* | 4.78 |
|  | *ACYP2* | 3.67 |
|  | *FUNDC1* | 3.44 |
|  | *COX7B* | 2.00 |
|  | *MEST* | 1.89 |
|  | *DPCD* | 1.78 |
|  | *TCP10L* | 1.78 |
|  | *MCRS1* | 1.78 |
|  | *LINC0014* | 1.67 |
|  | *METTL5* | 1.56 |
|  | *IL11* | 1.33 |
|  | *SSBP3* | 1.33 |
|  | *ELF5* | 1.11 |
|  | *TMEM185A* | 1.00 |
|  | *RXRB* | 0.89 |
|  | *SVIL* | 0.78 |
|  | *STAC2* | 0.78 |
|  | *VENTX* | 0.56 |
|  | *COX20* | 0.56 |
|  | *LOC730098* | 0.56 |
|  | *UQCRB* | 0.44 |
|  | *SERP2* | 0.33 |
|  | *CDK6* | 0.33 |
|  | *GOLGA6D* | 0.22 |
|  | *CLPS* | 0.11 |
| ***C9orf72-related familial ALS or sporadic ALS sample*** | | |
|  | *HOXD9* | 9.56 |
|  | *C9orf72* | 9.33 |
|  | *GP9* | 5.78 |
|  | *CFAP57* | 5.67 |
|  | *CRYZL1* | 1.56 |
|  | *PWAR6* | 0.78 |
|  | *ELOA3C* | 0.67 |
|  | *SVIL* | 0.67 |
|  | *OTP* | 0.56 |
| **Motor neuron classifiers** | | |
| ***Sporadic ALS or healthy sample*** | | |
|  | *DDB1* | 8.67 |
|  | *MIR16* | 6.78 |
|  | *CCDC85B* | 6.44 |
|  | *GJA5* | 5.00 |
|  | *ELAVL3* | 2.33 |
|  | *KLHL18* | 2.00 |
|  | *NQO1* | 1.67 |
